# The impact of elderly digital literacy on subjective wellbeing: a moderated mediation model

**DOI:** 10.3389/fpsyg.2026.1859788

**Published:** 2026-07-17

**Authors:** Jihui Liu, Yan Qiao, Yuanyuan Shao, Chuhan Fu, Tongxin Wang, Quanquan Guo, Xuan Zhao

**Affiliations:** College of Humanities and Social Sciences, Hebei Agricultural University, Baoding, China

**Keywords:** acceptance of artificial intelligence-based elderly care services, digital literacy, moderated mediation model, self-efficacy, subjective wellbeing

## Abstract

**Introduction:**

This study investigates how digital literacy influences subjective wellbeing among older adults, focusing on the mediating role of self-efficacy and the moderating role of acceptance of artificial intelligence (AI)-based elderly care services. The aim is to provide empirical evidence for enhancing older adults’ subjective wellbeing and optimizing intelligent elderly care services.

**Methods:**

A survey was conducted with 425 older adults using validated scales measuring digital literacy, self-efficacy, acceptance of AI-based elderly care services, and subjective wellbeing. Data were analyzed using SPSS and Mplus, including descriptive statistics, correlation analysis, mediation tests, moderation tests, and moderated mediation analysis.

**Results:**

The results showed that digital literacy positively affected subjective wellbeing (*r* = 0.154, *p* < 0.01); digital literacy positively affected self-efficacy (*r* = 0.372, *p* < 0.01); self-efficacy positively affected subjective wellbeing (*r* = 0.152, *p* < 0.01). self-efficacy partially mediated the relationship between digital literacy and subjective wellbeing (indirect effect β = 0.041, *p* < 0.01). Acceptance of AI-based elderly care services positively moderated the relationship between digital literacy and self-efficacy (β = 0.164, *p* < 0.01). The moderated mediation effect was significant (index of moderated mediation = 0.269; 95% bootstrap confidence interval excluded zero), and the mediating effect of self-efficacy was stronger for older adults with higher acceptance of AI-based services.

**Discussion:**

These findings suggest that in an aging society, efforts should be made to enhance older adults’ digital literacy and foster positive acceptance of AI-based elderly care services, thereby improving subjective wellbeing through increased self-efficacy. This study provides a reference for the formulation of intelligent elderly care policies.

## 1 Introduction

Subjective wellbeing is an overall evaluation of an individual’s quality of life based on their own standards, encompassing two dimensions: cognitive judgment and emotional experience. It is an important indicator of mental health among older adults (Xing, 2002). Currently, China is accelerating its transition into a deeply aging society. By the end of 2023, the population aged 60 and above reached 297 million, accounting for 21.1% of the total population (Niu and Cao, 2024). In the context of population aging, enhancing the quality of life and subjective wellbeing of older adults has become a major societal concern. Simultaneously, digital technology has become deeply integrated into residents’ daily lives, ranging from mobile payments to online healthcare, from social communication to information acquisition. Digitalization has deeply embedded itself in the operation mode of modern society. However, due to cognitive changes and a slower pace of learning and adaptation, older adults lag significantly behind in accessing and using digital technology. This “digital divide” prevents many older adults from reaping the benefits of digitalization, restricts their social participation and daily convenience, and may negatively affect their subjective wellbeing.

In response, researchers have begun to examine the relationship between digital literacy among older adults and their subjective wellbeing. Digital literacy refers to an individual’s ability to acquire, use, create, and safeguard information in a digital environment (Bandura, 1986). Existing studies indicate that improved digital literacy helps older adults enhance their autonomy in daily life and improve their emotional state (Liu and Xie, 2021). Nevertheless, most current research focuses on the direct correlation between the two, with insufficient attention to the underlying mediating mechanisms.

Social Cognitive Theory provides a suitable theoretical framework for addressing this gap. The theory posits that individual behavior, environment, and personal cognition interact reciprocally. And self-efficacy, which refers to an individual’s belief in their ability to complete a specific task, is the crucial link between cognition and behavior (Bandura, 1986). In the digital context, self-efficacy manifests as older adults’ confidence in mastering digital technology, which determines whether they persist or give up when encountering technical difficulties. Existing research has shown that self-efficacy plays a significant mediating role in health behaviors and quality of life among older adults (Tang et al., 2006). While current studies have preliminarily revealed relationships between self-efficacy, digital literacy, and subjective wellbeing in older adults, they lack detailed mechanistic analysis, overlook contextual factors, and neglect the emerging context of “the rise of artificial intelligence services” in the aging society.

In recent years, with advancements in science and technology, artificial intelligence has been increasingly applied in elderly care services. Intelligent health monitoring devices, voice assistants, companion robots, and other AI products have gradually entered the homes of older adults (Cui and Liu, 2022). However, acceptance of AI technology varies considerably among older adults. This variation is related not only to the perceived ease of use of the technology itself but also to whether older adults view AI technology as a reliable environmental resource (Xu and Su, 2021). Social Cognitive Theory suggests that a supportive technological environment can foster positive cognition (Bandura, 1986). This implies that acceptance of AI-based elderly care services may serve as a situational factor that moderates the efficiency with which digital literacy translates into self-efficacy. Building on this analysis, this study constructs a moderated mediation model grounded in social cognitive theory to examine how digital literacy mediates the impact of self-efficacy on subjective wellbeing, and how acceptance of AI-based care services moderates this mediation effect. The findings aim to clarify the contextual conditions under which self-efficacy exerts its influence and provide a theoretical foundation for tiered interventions in intelligent elderly care policies.

## 2 Theory and hypotheses

### 2.1 Digital literacy and subjective wellbeing

Subjective wellbeing refers to an individual’s comprehensive evaluation of their quality of life based on self-defined criteria, encompassing both cognitive assessment and emotional experience (Xing, 2002), including both rational evaluations of life satisfaction and a balance between positive and negative emotions (Niu and Cao, 2024). Among the elderly population, subjective wellbeing serves as a key indicator of mental health, quality of life (Tang et al., 2006), and successful aging, reflecting the overall psychological state of older adults in facing physiological decline, changes in social roles, and environmental adaptation processes.

Social Cognitive Theory suggests that an individual’s cognitive ability directly affects their adaptability to the environment and behavioral outcomes (Bandura, 1986). In recent years, amid the convergence of digitalization and population aging, digital literacy has emerged as a key factor affecting elderly individuals’ subjective wellbeing. Digital literacy, as an important cognitive ability, enables older adults to independently complete online tasks, obtain health information, and maintain social connections, thereby enhancing their sense of control over life. This enhanced sense of control translates into positive life experiences, which are a core source of subjective wellbeing (Xing, 2002). Conversely, insufficient digital literacy may cause older adults to encounter persistent difficulties in a digital society, leading to feelings of helplessness and alienation. Such negative emotions can weaken their subjective wellbeing. Based on survey data, Niu and Cao (2024) found that the five dimensions of digital literacy, namely physical access, information search, communication and collaboration, data security, and content creation, are all significantly positively correlated with the subjective wellbeing among older adults (Niu and Cao, 2024). Similarly, Liu and Xie (2021) confirmed that improvements in digital skills enhance the subjective wellbeing of older adults (Liu and Xie, 2021). Therefore, digital literacy can be considered an important predictor of subjective wellbeing in this population.

Based on the above analysis, this study proposes the following hypothesis:

*H1:* Digital literacy positively influences subjective wellbeing among older adults.

### 2.2 The mediating role of self-efficacy

One of the main sources of self-efficacy is the successful experiences an individual has encountered (Bandura, 1997). When older adults acquire digital operation skills through learning and are able to independently complete tasks that previously required assistance from others, these successful experiences strengthen their internal belief in their own capabilities. Improved digital literacy means that older adults have accumulated more successful experiences in using digital technology, and these experiences continuously reinforce their positive self-evaluation. Conversely, older adults who lack digital literacy frequently encounter operational failures, and such failure experiences can weaken their self-efficacy. Geng and Fu (2023) found that older adults’ network efficacy varies significantly by age, educational level, and internet usage ability (Geng and Fu, 2023), indicating that digital ability is closely related to efficacy. Pu and Huang (2023) further demonstrated through meta-analysis that information literacy is significantly positively correlated with information literacy self-efficacy (Pu and Huang, 2023). Therefore, improving digital literacy is an important pathway to enhancing self-efficacy among older adults.

Based on the above analysis, this study proposes the following hypothesis:

*H2:* Digital literacy positively affects self-efficacy among older adults.

self-efficacy has a broad impact on psychological adaptation by influencing an individual’s goal setting, coping strategies, and emotional state (Bandura, 1997). Older adults with higher self-efficacy are more willing to adopt positive coping methods when facing life problems, believing in their ability to overcome difficulties and maintaining an optimistic emotional state. This positive cognitive tendency generalizes to various aspects of life, enabling them to make more positive evaluations of their own lives. Older adults with low self-efficacy are prone to feelings of helplessness and anxiety when facing difficulties, and these negative emotions directly reduce their subjective wellbeing (Tang et al., 2006). Tong (2004) found that general self-efficacy has a significant positive predictive effect on subjective wellbeing (Tong, 2004). Gao et al. (2017) further confirmed through dominance analysis that self-efficacy contributes significantly to predicting subjective wellbeing among older adults, alongside psychological resilience and loneliness (Gao et al., 2017). Therefore, self-efficacy is a key psychological resource for maintaining a positive mindset and happy experiences in later life.

Based on the above analysis, this study proposes the following hypothesis:

*H3:* self-efficacy positively affects subjective wellbeing among older adults.

As an internal psychological resource possessed by individuals, self-efficacy plays a mediating role in the influence of digital literacy on subjective wellbeing. According to Social Cognitive Theory, cognitive ability (digital literacy) affects an individual’s belief in their own ability (self-efficacy), which in turn affects their emotional experience and behavioral outcomes (subjective wellbeing) (Bandura, 1986). Specifically, digital literacy not only directly brings convenience to older adults’ daily lives but, more importantly, enhances their self-efficacy, thereby building their confidence in coping with challenges in the digital sphere. This confidence extends from the domain of digital use to other aspects of life, enabling older adults to form a more positive assessment of their overall wellbeing. When self-efficacy is lacking, even if older adults possess certain digital skills, they may be reluctant to try or may give up easily due to a lack of confidence, thus failing to convert digital capabilities into a happy experience. Tang et al. (2010) also pointed out in their review that emotional regulation self-efficacy influences subjective wellbeing through interpersonal efficacy (Tang et al., 2010). Thus, the effect of digital literacy on subjective wellbeing is largely achieved through the mediating variable of self-efficacy.

Based on the above analysis, this study proposes the following hypothesis:

*H4:* self-efficacy mediates the relationship between digital literacy and subjective wellbeing among older adults.

### 2.3 The moderating effect of acceptance of artificial intelligence-based elderly care services

Artificial intelligence-based elderly care services represent a novel service model developed through the deep integration of AI technology with elderly care services (Xu and Su, 2021). Targeting seniors, these services leverage smart devices, big data platforms, and cloud computing, utilizing technologies such as speech recognition, image recognition, natural language processing, expert systems, and deep learning to provide services including daily care, medical assistance, emotional support, and emergency aid (Suan and Cao, 2019), characterized by intelligence, real-time responsiveness, interactivity, and round-the-clock coverage (Cui and Liu, 2022). In this study, “acceptance of artificial intelligence-based elderly care services” refers to seniors’ willingness and acceptance of utilizing these intelligent technologies and devices for receiving care services.

Within the framework of Social Cognitive Theory, acceptance of artificial intelligence (AI)-based elderly care services can be regarded as an important environmental factor. This theory clearly states that an individual’s cognition and behavior are regulated by environmental factors, and that a supportive technological environment can facilitate the development of positive cognition (Bandura, 1986). AI acceptance, as an environmental variable, influencing the relationship between digital literacy and self-efficacy by affecting the sources of self-efficacy, namely through experiencing successful experiences. Older adults with a high level of AI acceptance are more willing to try using AI products. These successful usage experiences provide ample personal success experiences, thereby enhancing the effectiveness of digital literacy in improving self-efficacy. In contrast, for older adults with low AI acceptance, even if they possess a certain level of digital literacy, they tend to avoid using AI products and consequently have difficulty obtaining successful experiences. As a result, the efficiency of converting digital literacy into self-efficacy is significantly reduced. Cui and Liu (2022) noted that AI-based elderly care services deliver precise services to older adults through intelligent devices, which helps improve their quality of life . Xu and Su (2021) also emphasized that improving older adults’ ability to use AI devices is an important measure. Thus, AI acceptance constitutes the contextual boundary for the influence of digital literacy on self-efficacy.

Based on the above analysis, this study proposes the following hypothesis:

*H5:* Acceptance of AI-based elderly care services moderates the relationship between digital literacy and self-efficacy among older adults. Specifically, the higher the acceptance, the stronger the positive effect of digital literacy on self-efficacy, and vice versa.

Furthermore, acceptance of AI-based elderly care services not only moderates the relationship between digital literacy and self-efficacy but also influences the strength of the mediating role of self-efficacy in the overall pathway. According to Conservation of Resources Theory, individuals are more likely to convert internal psychological resources into actual behaviors when they have sufficient external resource support (Bandura, 1986). When AI acceptance is high, the environment provides favorable technological support for older adults. The self-efficacy stimulated by improved digital literacy can be more smoothly transformed into actual usage behaviors and life improvements, thereby generating a stronger positive effect on subjective wellbeing. In other words, the mediating effect of self-efficacy is amplified. Conversely, in a low AI acceptance environment, even if self-efficacy has increased, the lack of external technical support makes it difficult to effectively translate self-efficacy into a happy experience, thus weakening the mediating effect. Cui and Liu (2022) reviewed that intelligent elderly care services offer advantages such as intelligence and interactivity, providing new channels of support for older adults. Li et al. (2023) also found that in the process of bridging the digital divide, external environmental factors influence wellbeing through cognitive variables. This further supports the theoretical logic that AI acceptance, as a contextual variable, moderates the mediating effect.

Based on the above analysis, this study proposes the following hypothesis:

*H6:* Acceptance of AI-based elderly care services moderates the mediating effect of self-efficacy in the relationship between digital literacy and subjective wellbeing among older adults. Specifically, the higher the acceptance, the stronger the mediating effect of self-efficacy, and vice versa.

### 2.4 Summary of research model and hypotheses

**FIGURE 1:**
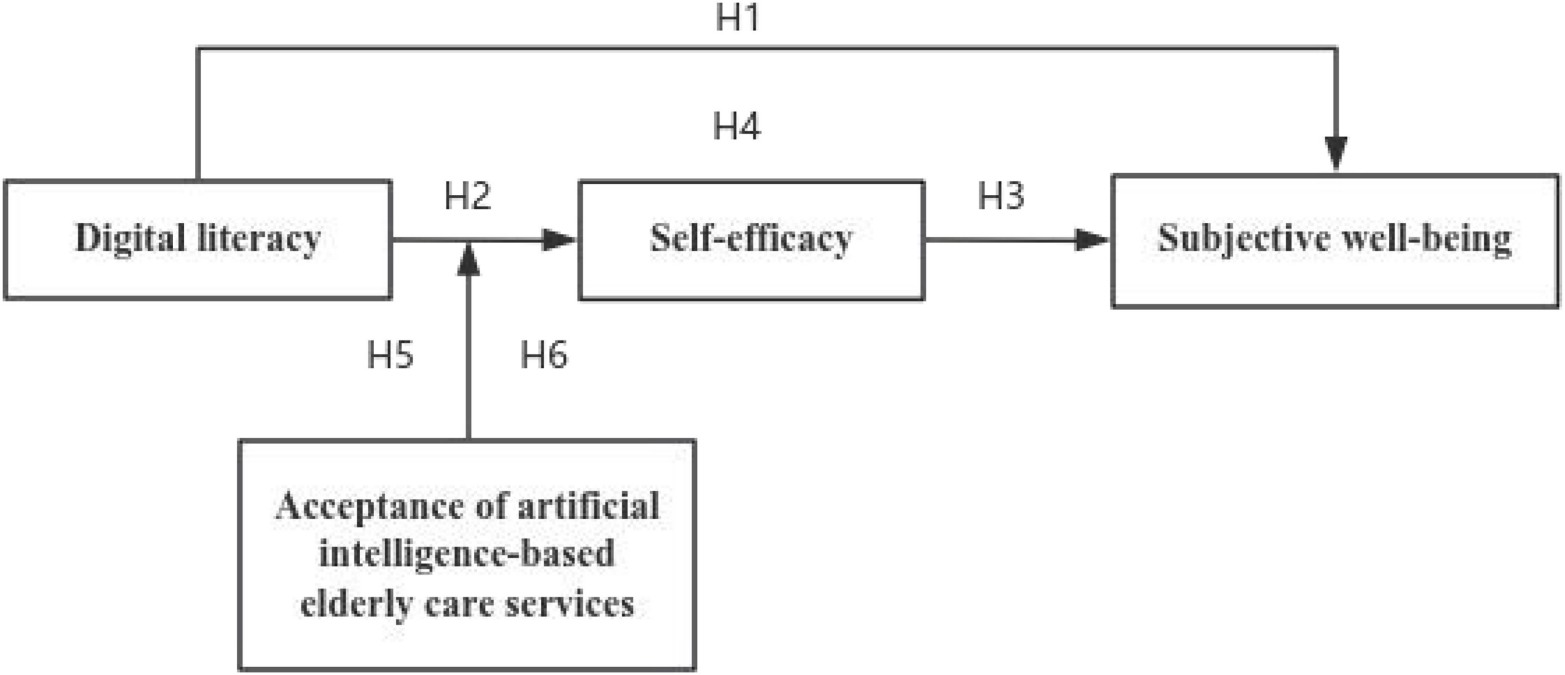
Theoretical model.

This study focused on older adults aged 60–75 years. Based on Social Cognitive Theory, a moderated mediation model was constructed. In this model, digital literacy served as the independent variable, subjective wellbeing as the dependent variable, self-efficacy as the mediating variable, and acceptance of artificial intelligence (AI)-based elderly care services as the moderating variable. The research model is presented in Figure 1.

Based on the research model constructed according to Social Cognitive Theory, this study proposes hypotheses regarding the relationships among four variables: digital literacy, subjective wellbeing, self-efficacy, and acceptance of artificial intelligence (AI)-based elderly care services. The specific hypotheses are as follows in Table 1.

**TABLE 1:** Summary of assumptions.

Sequence number	Theoretical hypothesis
H1	Digital literacy positively influences subjective wellbeing among older adults.
H2	Digital literacy positively affects self-efficacy among older adults.
H3	Self-efficacy positively affects subjective wellbeing among older adults.
H4	Self-efficacy mediates the relationship between digital literacy and subjective wellbeing among older adults.
H5	Acceptance of AI-based elderly care services moderates the relationship between digital literacy and self-efficacy among older adults. Specifically, the higher the acceptance, the stronger the positive effect of digital literacy on self-efficacy, and vice versa.
H6	Acceptance of AI-based elderly care services moderates the mediating effect of self-efficacy in the relationship between digital literacy and subjective wellbeing among older adults. Specifically, the higher the acceptance, the stronger the mediating effect of self-efficacy, and vice versa.

## 3 Research method

### 3.1 Data sources

This study employed a questionnaire survey method. The research area was the Beijing-Tianjin-Hebei region, and participants were community-dwelling older adults aged 60–75 years. The Beijing-Tianjin-Hebei region presents a unique ique communityunitymoderates the mediating effect of self-efficacy in the relationship btal resources, Tianjin has a mixed urban-rural layout, and Hebei serves as a major provider of elderly care but lags in digital infrastructure. Older adults living in communities across these three regions face distinct pressures regarding digital integration. Furthermore, the collaborative elderly care policy within the Beijing-Tianjin-Hebei region and internal regional mobility add further complexity.

This study conducted large-scale data collection using quantitative questionnaires, which included scales for digital literacy, acceptance of artificial intelligence (AI)-based elderly care services, self-efficacy, and subjective wellbeing. To reduce common method bias, data were collected in two waves, with a 1-month interval between them. In the first wave, demographic variables, independent variables (digital literacy), and the mediating variable (self-efficacy) were measured. In the second wave, the moderating variable (acceptance of AI-based elderly care services) and the dependent variable (subjective wellbeing) were measured. This temporal separation helped avoid biases caused by habitual thinking during questionnaire completion, thereby improving the authenticity and accuracy of the responses. In the first wave, 215 electronic questionnaires were distributed, and 200 valid questionnaires were returned, yielding an effective response rate of 93.02%. One month later, the research team distributed another 250 questionnaires, and 225 valid questionnaires were returned, with an effective response rate of 90.0%. In total, 425 valid questionnaires were collected for this study. Among them, there were 122 samples from Beijing, 128 from Tianjin, and 175 from Hebei. The sample distribution in these three regions was basically even.

### 3.2 Measures

This study focused on four core variables: digital literacy of older adults, acceptance of artificial intelligence (AI)-based elderly care services, self-efficacy, and subjective wellbeing. Considering the appropriateness of the scales for the Chinese context, we selected relevant scales from authoritative academic journals. Prior to use, translation and back-translation procedures were carried out to ensure semantic equivalence between the Chinese and English versions, thereby ensuring the scientific rigor and adaptability of the measurement tools.

Since the selected scales were originally developed in English, this study strictly followed the translation-back-translation procedure proposed by Brislin (1970) to achieve semantic, conceptual, and cultural equivalence between the Chinese and original versions. Specifically, two mastercally, inese and original versionsal equivalence nglish, this study strto Chinese, producing two draft translations. Through comparison and discussion of these drafts, an initial Chinese version was formed. Subsequently, two English major students independently translated the initial Chinese version back into English. The back-translated versions were then compared with the original English version item by item to identify semantic discrepancies, and the Chinese wording was revised accordingly to determine the final scale.

To accurately estimate the net effect of digital literacy on subjective wellbeing of older adults, this study controlled for variables such as gender, education level, and personal monthly income. Existing research has shown that these variables are confounders affecting older adults was formed. wellbeing. Gender is significantly correlated with subjective wellbeing (Peng et al., 2026); Cho (2025) confirmed that education level and income are significant predictors of subjective health status; another study also found that income is significantly positively correlated with older adults’ subjective wellbeing (Meng and Wu, 2025). Therefore, including gender, education level, and personal monthly income as control variables helps eliminate their interference and accurately estimate the net effect of digital literacy.

To measure digital literacy of older adults, this study used the scale developed by Qu et al. (2025). Representative items include: er adults, this study used the scale developed by net effect of digital literacy.bsequently, ve response rate of 90.0 authenticity and accuracy of the responses. n efforts,” and “I have a positive attitude toward learning digital technology.igital technology.e:-point Likert scale, with higher scores indicating higher digital literacy.

The mediating variable in this study is self-efficacy. Consistent with the research topic, we used the self-efficacy scale developed by Schwarzer et al. (1997) . This scale has demonstrated good reliability and validity in prior research. It uses a 5-point Likert scale. Representative items include: lude: lork hard to learn digital technology, I will always be able to solve problems encountered when using electronic devicesty in prior research. It uses a 5ct of digital literacy.bsequently, ve responto learn and apply the digital functions I want.n

To measure acceptance of AI-based elderly care services, this study adopted the scale developed by Venkatesh et al. (2003). Representative items include: vel-based elderly care services make my daily life more convenientloped by -based elderly care services improve my health level.”

The dependent variable was subjective wellbeing. This study used the Index of wellbeing compiled by Campbell et al. (1976). The scale was scored on a 5-point Likert scale, with higher scores indicating stronger subjective wellbeing. The authors made adaptive modifications to the scale for this study and ultimately formed the final questionnaire.

### 3.3 Data analysis

This study used Mplus 8.0 and SPSS 26.0 to analyze the questionnaire data. Specifically, SPSS was employed to conduct descriptive analyses of control variables (e.g., gender, age, and educational background), as well as correlation analyses and reliability tests for all study variables. Mplus was used to perform validity tests through model comparison and to test the research hypotheses.

## 4 Results

### 4.1 Descriptive statistics

Among the 425 valid questionnaires, 209 participants were male (49.2%) and 216 were female (50.8%). Regarding educational attainment, 57 participants (13.4%) had no formal education or completed only junior high school or below; 142 participants (33.4%) had completed senior high school or equivalent (including vocational education); 177 participants (41.6%) held a college degree; and 49 participants (11.5%) held a bachelordegree; and 49 parti

In terms of personal monthly income, 52 participants (12.2%) reported no income; 141 participants (33.2%) reported an income of 0 particyuan (including 3,442 yuan); 143 participants (33.6%) reported an income of 3,442–3,615 yuan (including 3,615 yuan); and 89 participants (20.9%) reported an income above 3,615 yuan (Table 2).

**TABLE 2:** Demographic description.

Name	Options	Frequencym	Percentage (%)
Sex	Female	209	49.2
	Male	216	50.8
Education	Below junior high school	57	13.4
	Junior high school and senior high school (including vocational school)	142	33.4
	Junior college	177	41.6
	Bachelor’s degree and above	49	11.5
Personal monthly income	No income	52	12.2
	0-3,442 yuan (including 3442 yuan)	141	33.2
	3,442-3,615 yuan (including 3,615 yuan)	143	33.6
	More than 3,615 yuan	89	20.9

### 4.2 Correlations

As shown in Table 3, educational level and personal monthly income were significantly correlated with older adultsional school) education)-efficacy, and acceptance of AI-based elderly care services. Personal monthly income was also significantly correlated with older adultsl school) eduwellbeing. Furthermore, digital literacy was significantly positively correlated with subjective wellbeing (*r* = 0.154) and with self-efficacy (*r* = 0.372). Self-efficacy was significantly positively correlated with subjective wellbeing (*r* = 0.152). These results provide support for Hypotheses 1, 2, and 3.

**TABLE 3:** Correlations.

Variables	*M*	SD	1	2	3	4	5	6	7
1. Sex	1.51	0.501	1	1	1	1	1	1	1
2. Education	2.51	0.866	-0.048						
3. Personal monthly income	2.63	0.948	-0.033	0.638**					
4. Digital literacy	3.03	0.914	-0.025	0.567**	0.687**				
5. Self-efficacy	3.18	0.824	-0.027	0.196**	0.307**	0.372**			
6. AI elderly care acceptance	3.02	0.857	-0.056	0.240**	0.324**	0.467**	0.309**		
7. Subjective wellbeing	2.85	0.805	-0.043	0.073	0.149**	0.154**	0.152**	0.314**	

**p < 0.01.

### 4.3 Reliability analysis

Reliability analysis refers to the consistency, stability, and dependability of the measurements obtained. Internal consistency is commonly used to indicate the reliability level of a scale. In this study, reliability was assessed by calculating Cronbach’s α coefficient for each scale. A larger Cronbach’s α indicates stronger internal consistency among the items and higher reliability of the scale. A Cronbach’s α> 0.7 is generally considered acceptable.

The results are presented in Table 4. The Cronbach’s α coefficients for digital literacy, self-efficacy, acceptance of AI-based elderly care services, and subjective wellbeing were 0.913, 0.963, 0.911, and 0.905, respectively. All values exceeded 0.7, indicating good internal consistency of the scales and high reliability of the measurement results.

**TABLE 4:** Reliability analysis of various variables.

Variable	Cronbach’s alpha
Digital literacy	0.913
Subjective wellbeing	0.963
Acceptance of artificial intelligence-based elderly care services	0.911
Self-efficacy	0.905

### 4.4 Confirmatory factor analysis

Confirmatory factor analysis (CFA) was conducted using Mplus 8.0 to test whether the hypothesized theoretical model was consistent with the observed data. Four competing models were specified: the baseline (four-factor) model, a three-factor model, a two-factor model, and a one-factor model. Model fit was evaluated using the χ^2^ test, the ratio χ^2^/df, RMSEA, CFI, TLI, and SRMR. In addition, the significance of factor loadings was examined to confirm the explanatory power and reliability of each variable’s correspondence with its factor. This procedure enhanced the construct validity and predictive power of the model.

The results (shown in Table 5) indicated that the three-factor, two-factor, and one-factor models all exhibited certain fit problems, suggesting a degree of mismatch with the data. To determine which model best explained the observed data, we examined the baseline model, which included the four variables: older adults’ digital literacy, self-efficacy, acceptance of AI-based elderly care services, and subjective wellbeing.

**TABLE 5:** Common method deviation.

Model	χ ^2^	df	χ ^2^/df	CFI	TLI	RMSEA	SRMR
Baseline model (DI; SE; AIA; SWB)	1022.222	896	1.14	0.990	0.989	0.018	0.042
Three factor model (DI; SE+AIA;SWB)	2941.812	899	3.27	0.831	0.822	0.073	0.107
Two factor model (DI; SE+AIA+SWB)	4893.480	901	5.43	0.669	0.652	0.102	0.141
Single factor model (DI+SE+AIA+SWB)	6439.529	902	7.14	0.541	0.518	0.120	0.153

*N* = 425. DI, Digital Literacy of the Elderly; SE, Self-Efficacy; AIA, Acceptance of AI-based Elderly Care Services; SWB, Subjective Wellbeing. CFI, Comparative Fit Index; TLI, Tucker–Lewis Index; RMSEA, Root Mean Square Error of Approximation; SRMR, Standardized Root Mean Square Residual.

The baseline model demonstrated excellent fit: χ^2^/df = 1.14 ( < 3), CFI = 0.990 ( > 0.9), TLI = 0.989 ( > 0.9), RMSEA = 0.018 ( < 0.08), and SRMR = 0.042 ( < 0.08). All fit indices met the recommended criteria, indicating that the baseline model was the most suitable for explaining the observed data and possessed good construct validity.

The three-factor model combined self-efficacy and acceptance of AI-based elderly care services into one factor, with digital literacy and subjective wellbeing as separate factors. Its fit was poorer: χ^2^/df = 3.27 ( > 3), CFI = 0.831, TLI = 0.822, RMSEA = 0.073, SRMR = 0.107 ( > 0.08).

The two-factor model combined self-efficacy, acceptance of AI-based elderly care services, and subjective wellbeing into one factor, with digital literacy as the other factor. Fit indices were: χ^2^/df = 5.43 ( > 3), CFI = 0.669, TLI = 0.652 (both < 0.8), RMSEA = 0.102 ( > 0.08), SRMR = 0.141 ( > 0.08), indicating poor fit.

The one-factor model combined all four variables into a single factor. Fit indices were: χ^2^/df = 7.14, CFI = 0.541, TLI = 0.518, RMSEA = 0.120, SRMR = 0.153, all indicating unacceptable fit.

In summary, the baseline (four-factor) model showed the best fit, confirming that the data have good construct validity and that the four variables are empirically distinct.

### 4.5 Common method bias test

To assess the potential influence of common method bias, the Harman single-factor test was conducted on the items of the four scales using the collected data. The results (shown in Table 6) indicated that the variance explained by the first unrotated factor was only 26.615%, which is below the commonly recommended threshold of 40%. Therefore, common method bias did not pose a serious threat to the validity of the study, and its influence on the research data was within an acceptable range.

**TABLE 6:** Common method deviation.

Ingredient	Initial eigenvalue			Extract the sum of squared loads					
Variables	Total	Variance percentage	Accumulated%	Total	Variance percentage	Accumulated%		Total	Variance percentage
1	14.125	32.101	32.101	14.125	32.101	32.101	11.711	26.615	26.615
2	5.923	13.461	45.562	5.923	13.461	45.562	5.217	11.857	38.472
3	4.056	9.219	54.781	4.056	9.219	54.781	5.030	11.433	49.905
4	2.457	5.583	60.364	2.457	5.583	60.364	4.602	10.460	60.364
…	…	…	…	…	…	…	…	…	…
44	0.081	0.185	100.000						

### 4.6 Analysis of the mediating effect of self-efficacy

This study hypothesizes that self-efficacy plays a mediating role between digital literacy and subjective wellbeing among the elderly (Hypothesis 4). The mediating effect can be classified into two types: complete mediation and partial mediation. Complete mediation refers to the situation where the influence of the predictor variable on the dependent variable is completely achieved through the mediating variable, meaning that after including the mediating variable, the direct effect of the predictor variable on the dependent variable is not significant, and the effect of the predictor variable on the dependent variable is entirely dependent on the role of the mediating variable. That is, when the direct effect is not significant but the indirect effect is significant, the mediation result is a complete mediation effect. Partial mediation is where the influence of the predictor variable on the dependent variable is partially transmitted through the mediating variable, meaning that after including the mediating variable, the direct effect of the predictor variable on the dependent variable is still significant, and the predictor variable not only indirectly affects the dependent variable through the mediating variable but also directly affects it. That is, when both the direct effect and the indirect effect are significant, the mediation result is a partial mediation effect.

To verify that self-efficacy plays a mediating role between digital literacy and subjective wellbeing among the elderly, this study explored the relationships between digital literacy and subjective wellbeing, digital literacy and self-efficacy, and self-efficacy and subjective wellbeing, respectively.

The research results (as shown in Table 7) indicate that in the path “elderly digital literacy → subjective wellbeing,” denoted as a, the direct effect coefficient is 0.437 (*p* < 0.01), the standard error is 0.055, and the 97.5% BootCI confidence interval is (0.327, 0.544). In the path “elderly digital literacy → self-efficacy,” denoted as b, the direct effect coefficient is 0.360 (*p* < 0.01), the standard error is 0.051, and the 97.5% BootCI confidence interval is (0.258, 0.457). In the path “self-efficacy → subjective wellbeing,” denoted as c, the direct effect coefficient is 0.114 (*p* < 0.01), the standard error is 0.053, and the 97.5% BootCI confidence interval is (0.012, 0.220).

**TABLE 7:** Test of mediating effect.

Item	Estimate	S.E.	97.5% BootCI	
Digital literacy → subjective wellbeing	0.437**	0.055	0.327	0.544
Digital literacy → self-efficacy	0.360**	0.051	0.258	0.457
Self-efficacy → subjective wellbeing	0.114**	0.053	0.012	0.220
Digital literacy → self-efficacy → subjective wellbeing	0.041** (Indirect effect);	0.020	0.005	0.085
Digital literacy → self-efficacy → subjective wellbeing	0.478** (Total effect)	0.051	0.377	0.578

**p < 0.01.

In the mediating path “elderly digital literacy → self-efficacy → subjective wellbeing,” which is b*c, the indirect effect value is 0.041 (*p* < 0.01), the standard error is 0.020, and the 97.5% BootCI confidence interval is (0.005, 0.085), which does not include 0, indicating that the indirect effect is significant. The total effect is 0.478 (*p* < 0.01), the standard deviation is 0.051, and the 97.5% BootCI confidence interval is (0.377, 0.578), which does not include 0, indicating that the total effect is significant.

In conclusion, both the direct effect and the indirect effect are significant; thus, the mediating path is established and is a partial mediation. Therefore, Hypothesis 4 is verified: self-efficacy plays a mediating role between elderly digital literacy and life happiness.

### 4.7 Analysis of the moderating effect of acceptance of artificial intelligence-based elderly care services

This study hypothesizes that the acceptance of artificial intelligence-based elderly care services has a moderating effect on the relationship between digital literacy and self-efficacy among the elderly. Specifically, the higher the acceptance of artificial intelligence-based elderly care services, the stronger the positive impact of digital literacy on self-efficacy, and vice versa (H5). The moderating effect is primarily tested by examining whether the confidence interval of the interaction term includes zero. If zero is not included, the path is significant, indicating a significant moderating effect.

The analysis (as shown in Table 8) revealed that the interaction effect between digital literacy and acceptance of artificial intelligence-based elderly care services was significant and positive (β = 0.164, *p* < 0.01), with a confidence interval of (0.087, 0.262) that does not include zero. This indicates that the acceptance of artificial intelligence-based elderly care services positively moderates the impact of digital literacy on self-efficacy among the elderly. Therefore, Hypothesis H5 is supported.

**TABLE 8:** Analysis of moderating effects.

Variable	Self-efficacy(SE)			
	Estimate	S.E.	97.5% BootCI	
Intercepts	0.031	0.155	-0.273	0.336
Digital literacy(DI)	0.629**	0.036	0.558	0.699
Acceptance of artificial intelligence-based elderly care services(AIA)	0.174**	0.045	0.087	0.262
DI × AIA	0.164**	0.044	0.077	0.251

**p < 0.01.

**FIGURE 2:**
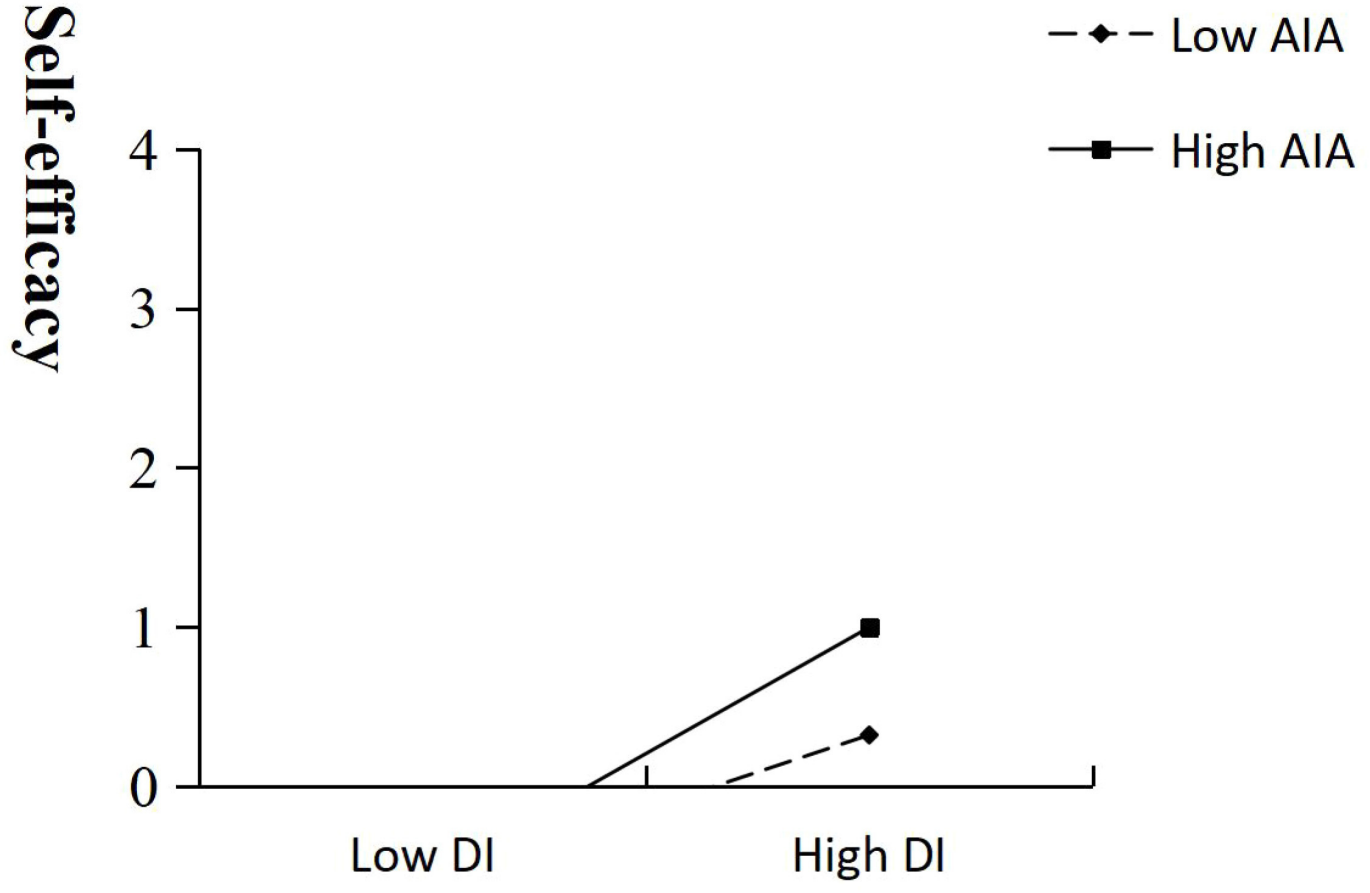
The moderating effect of the acceptance of artificial intelligence-based elderly care services between the digital literacy of the elderly and their self-efficacy.

To further verify the moderating effect of acceptance of artificial intelligence-based elderly care services, this study also employed the simple slope analysis method to examine the influence of different levels of acceptance on the relationship between digital literacy and self-efficacy among the elderly. As shown in Figure 2, at a low level of acceptance of artificial intelligence-based elderly care services, the effect of digital literacy on self-efficacy was significant (β = 0.465, *p* < 0.01). At a high level of acceptance (+1 SD), the positive effect of digital literacy on self-efficacy was also significant (β = 0.793, *p* < 0.01). Thus, the higher the acceptance of artificial intelligence-based elderly care services, the stronger the positive relationship between digital literacy and self-efficacy among the elderly. Therefore, Hypothesis H5 was again supported.

This study also hypothesizes that the acceptance of artificial intelligence-based elderly care services moderates the mediating role of self-efficacy between digital literacy and life happiness among the elderly. Specifically, the higher the acceptance of artificial intelligence-based elderly care services, the stronger the mediating effect of self-efficacy, and vice versa (H6). The analysis (as shown in Table 9) reveals that the interaction term between self-efficacy and acceptance of artificial intelligence-based elderly care services has a significant predictive effect on subjective wellbeing (β = 0.269, *p* < 0.01). This indicates that the mediating role of self-efficacy in the relationship between digital literacy and subjective wellbeing is moderated by the acceptance of artificial intelligence-based elderly care services. Therefore, Hypothesis H6 is supported.

**TABLE 9:** Analysis of moderating effects.

Variable	Subjective wellbeing(SWB)			
	Estimate	S.E.	97.5% BootCI	
Intercepts	0.170	0.125	-0.073	0.422
Digital literacy(DI)	0.215**	0.044	0.130	0.302
Subjective wellbeing(SE)	0.426**	0.042	0.343	0.510
Acceptance of artificial intelligence-based elderly care services(AIA)	0.136**	0.039	0.056	0.212
SE × AIA	0.269**	0.039	0.190	0.343

**p < 0.01.

The analysis of the magnitude of the mediating effect of self-efficacy at different levels of acceptance of artificial intelligence-based elderly care services (see Table 10) shows the following. When acceptance is low (M–1 SD), the indirect effect of digital literacy on subjective wellbeing through self-efficacy is significant, with an indirect effect value of 0.110, 97.5% CI = [0.026, 0.186]. When acceptance is at the mean (M), the indirect effect is significant, with a value of 0.298, 97.5% CI = [0.234, 0.358]. When acceptance is high (M + 1 SD), the indirect effect is significant, with a value of 0.485, 97.5% CI = [0.402, 0.560]. These results indicate that moderated mediation is established; that is, as the acceptance of artificial intelligence-based elderly care services increases, the indirect effect of digital literacy on subjective wellbeing through self-efficacy also increases. Therefore, Hypothesis H6 is supported.

**TABLE 10:** Indirect effects of self-efficacy at different levels of acceptance of AI-based elderly care services.

Acceptance of artificial intelligence-based elderly care services	Indirect effect value	S.E.	BootLLCI	BootULCI
Low (M-1SD)	0.110**	0.045	0.026	0.186
Mean (M)	0.298**	0.034	0.234	0.358
High (M+1SD)	0.485**	0.044	0.402	0.560

**p < 0.01.

## 5 Summary and discussion

### 5.1 Summary of hypothesis testing

This study, grounded in Social Cognitive Theory, constructed a moderated mediation model with self-efficacy as the mediating variable and acceptance of artificial intelligence (AI)-based elderly care services as the moderating variable. The model systematically examined the mechanism through which digital literacy affects subjective wellbeing among older adults. A total of 425 valid samples were collected via questionnaire surveys, and the hypotheses were tested using structural equation modeling and hierarchical regression analysis. The specific results are presented in Table 11.

**TABLE 11:** Assumption summary.

Sequence number	Theoretical hypothesis	Verification result
H1	Digital literacy positively influences subjective wellbeing among older adults.	Establishment
H2	Digital literacy positively affects self-efficacy among older adults.	Establishment
H3	Self-efficacy positively affects subjective wellbeing among older adults.	Establishment
H4	Self-efficacy mediates the relationship between digital literacy and subjective wellbeing among older adults.	Establishment
H5	Acceptance of AI-based elderly care services moderates the relationship between digital literacy and self-efficacy among older adults. Specifically, the higher the acceptance, the stronger the positive effect of digital literacy on self-efficacy, and vice versa.	Establishment
H6	Acceptance of AI-based elderly care services moderates the mediating effect of self-efficacy in the relationship between digital literacy and subjective wellbeing among older adults. Specifically, the higher the acceptance, the stronger the mediating effect of self-efficacy, and vice versa.	Establishment

### 5.2 Summary of research findings

Through empirical analysis, this study reached the following conclusions with theoretical significance and practical value.

First, digital literacy has a significant positive impact on the subjective wellbeing of older adults. The more digitally literate older adults are, the more capable they are of independently completing online tasks, obtaining health information, and maintaining social connections, thereby enhancing their sense of control over life and improving their subjective wellbeing. According to Social Cognitive Theory, an individually literate older adults are, the more capable they areto the environment and behavioral outcomes (Bandura, 1986). Digital literacy, as an important cognitive ability, helps older adults gain more autonomy and positive experiences in the digital society.

Second, digital literacy indirectly affects subjective wellbeing through self-efficacy, with self-efficacy playing a partial mediating role. Improved digital literacy enables older adults to accumulate successful experiences in digital usage, and such firsthand successful experiences are the core source of self-efficacy (Bandura, 1997). Older adults with high self-efficacy are more inclined to adopt positive coping strategies and maintain an optimistic emotional state, thereby achieving higher levels of happiness (Tang et al., 2006). This mediating path reveals the internal psychological mechanism through which digital capabilities are transformed into happiness experiences.

Third, the acceptance of AI-based elderly care services positively moderates the impact of digital literacy on self-efficacy and further moderates the mediating effect of self-efficacy. According to Social Cognitive Theory, a favorable technological environment can promote the formation of positive cognition (Bandura, 1986). Older adults with high acceptance of AI products view these products as reliable support resources and are more willing to try and use them, thereby obtaining more successful experiences and strengthening the enhancing effect of digital literacy on self-efficacy. At the same time, Conservation of Resources Theory suggests that when individuals have sufficient external resource support, they are more inclined to convert their internal psychological resources into actual behaviors (Li et al., 2023). A high AI acceptance environment provides favorable technical support for older adults, enabling the self-efficacy stimulated by digital literacy to be more smoothly transformed into actual improvements in life, thereby amplifying the happiness-enhancing effect.

Fourth, the moderated mediation model constructed in this study applies Social Cognitive Theory to the field of digital integration among older adults, expanding the theory Cognitivetory power at the intersection of aging and digitalization. The results show that enhancing digital literacy of older adults is a necessary condition, but the mediating role of self-efficacy and the contextual moderating effect of AI-based elderly care service acceptance should not be overlooked. This finding provides empirical references for the formulation of active aging policies and the design of adaptive digital services for older adults, suggesting that policymakers and service providers should focus not only on digital skills training but also on the improvement of psychological efficacy and the creation of a supportive technological environment.

### 5.3 Mechanism analysis and summary

From the perspective of social cognition theory, the impact of digital literacy on the subjective wellbeing of the elderly is not a simple direct effect, but is mediated through the internal psychological resource of self-efficacy, and is regulated by the environmental factor of the acceptance of artificial intelligence elderly care services. Specifically, the mechanism of its effect mainly manifests in the following three aspects.

First, self-efficacy serves as a “psychological converter” between digital ability and happiness experience. According to Bandura’s (1997) theory of self-efficacy, successful experiences firsthand are the most effective source of self-efficacy. When older adults acquire digital skills through learning and become capable of independently completing tasks that previously required assistance, these successes foster positive self-suggestions and expectations, leading to a sense of “self-fulfilling prophecy” (Geng and Fu, 2023). This belief is not limited to the field of digital use but will also generalize to other aspects of life, enabling the elderly to build confidence in their ability to cope with challenges, thereby enhancing overall wellbeing. Conversely, individuals lacking digital literacy frequently encounter operational failures during learning, which cultivates learned helplessness that undermines self-efficacy and negatively impacts subjective wellbeing (Zheng and Wang, 2024), trapping them in a vicious cycle of “helplessness -avoidance behavior-diminished efficacy.” Therefore, self-efficacy is the key psychological bridge that converts digital ability into happiness experience.

Second, the acceptance of artificial intelligence elderly care services, as an environmental resource, regulates the efficiency of the transformation of digital literacy into self-efficacy. Social cognition theory indicates that a good technological environment can promote the formation of positive cognition (Bandura, 1986). Elderly individuals with high AI acceptance view intelligent devices as reliable support resources and are more willing to actively try and use them. These successful usage experiences provide them with rich “experiences of successful experience,” thereby amplifying the positive impact of digital literacy on self-efficacy. Conversely, elderly individuals with low AI acceptance, even if they have certain digital abilities, will not obtain successful experiences due to avoidance of use, and the efficacy transformation of digital literacy is greatly reduced. This indicates that the improvement of the technological environment is not only providing tools but also an important condition for activating the internal psychological resources of the elderly.

Third, the regulated mediating effect reveals how environmental factors influence the final outcome through cognitive variables. According to resource conservation theory, individuals with ample external resource support are more inclined to translate internal psychological resources into concrete actions (Hobfoll, 1989). In environments with high AI acceptance, older adults not only exhibit higher self-efficacy but also benefit from sufficient external resource support, which motivates them to apply digital skills in practice (Peng and Hongliang, 2024), thereby significantly improving their quality of life and enhancing wellbeing. This mechanism indicates that enhancing the digital literacy of the elderly is important, but if there is a lack of a friendly technological environment and acceptance willingness, the psychological benefits will be difficult to be fully realized.

### 5.4 Research limitations and prospects

Although this study has initially revealed the mechanism by which digital literacy affects subjective wellbeing of older adults through self-efficacy and tested the moderating effect of AI acceptance, certain limitations remain. Future research should address these issues.

First, the cross-sectional nature of the research design has limitations. All variables were measured at the same time point, which can only explain the covariance between variables and cannot determine temporal sequence or dynamic evolution. There may be a bidirectional relationship between digital literacy and self-efficacy: an increase in digital literacy enhances self-efficacy, and older adults with high self-efficacy may also be more active in learning digital skills. Furthermore, the relationship between digital literacy and wellbeing may exhibit long-term cumulative effects or periodic fluctuations (Zhou et al., 2025). Future studies could adopt a longitudinal design to examine the dynamic relationships among the variables over time, allowing a more robust inference of causal direction.

Second, geographical limitations and structural biases of the sample may affect the generalizability of the conclusions. The sample in this study came mainly from the Beijing-Tianjin-Hebei region, with a majority of urban older adults; rural samples were relatively insufficient. Differences in digital infrastructure, elderly care policies, and cultural perceptions across regions may influence the magnitude of how digital literacy impacts wellbeing (Zhen et al., 2024). Future research should expand the sampling range to cover regions with different levels of economic development and different urban-rural characteristics, in order to test the cross-context consistency of the findings.

Third, the cultural adaptability of the measurement tools needs improvement. Although the scales used in this study underwent translation and back-translation, some items may not fully align with the language habits and cultural background of Chinese older adults. Future research could combine qualitative interviews to develop or revise measurement tools that are more suitable for the local context in China, thereby improving the ecological validity of the research.

Fourth, the simplicity of the model construction may lead to an incomplete understanding of the mechanism. Although this study controlled for variables such as gender, education, and income, it did not include other important factors that may affect the happiness of older adults, such as social support, health status, and living arrangements (Tang et al., 2006). In the future, the theoretical framework should be expanded to incorporate more contextual variables, and the heterogeneity of mechanisms across different groups (e.g., urban vs. rural, different age groups, different income levels) should be examined to better understand the relationship between digital integration of older adults and their sense of wellbeing.

## Data Availability

The original contributions presented in this study are included in the article/supplementary material, further inquiries can be directed to the corresponding author.
